# Changing hearts and minds: Results from a multi-country gender and sexual diversity training

**DOI:** 10.1371/journal.pone.0184484

**Published:** 2017-09-19

**Authors:** Tonia Poteat, Chulwoo Park, Diego Solares, John K. Williams, R. Cameron Wolf, Noah Metheny, Andrea Vazzano, Juan Dent, Ashley Gibbs, Bareng Aletta Sanny Nonyane, Nora Toiv

**Affiliations:** 1 Department of Epidemiology, Johns Hopkins Bloomberg School of Public Health, Baltimore, Maryland, United States of America; 2 Johns Hopkins Bloomberg School of Public Health, Baltimore, Maryland, United States of America; 3 Health Policy Project, The Palladium Group, Washington, District of Columbia, United States of America; 4 Division of Global HIV/TB, Centers for Disease Control and Prevention, Atlanta, Georgia, United States of America; 5 United States Agency for International Development, Washington, District of Columbia, United States of America; 6 Department of International Health, Johns Hopkins Bloomberg School of Public Health, Baltimore, Maryland, United States of America; 7 Office of the U.S. Global AIDS Coordinator, Washington, District of Columbia, United States of America; University of Toronto, CANADA

## Abstract

Engaging key populations, including gender and sexual minorities, is essential to meeting global targets for reducing new HIV infections and improving the HIV continuum of care. Negative attitudes toward gender and sexual minorities serve as a barrier to political will and effective programming for HIV health services. The President’s Emergency Plan for AIDS Relief (PEPFAR), established in 2003, provided Gender and Sexual Diversity Trainings for 2,825 participants including PEPFAR staff and program implementers, U.S. government staff, and local stakeholders in 38 countries. The outcomes of these one-day trainings were evaluated among a subset of participants using a mixed methods pre- and post-training study design. Findings suggest that sustainable decreases in negative attitudes toward gender and sexual minorities are achievable with a one-day training.

## Introduction

Gender norms and gender inequities contribute to HIV vulnerability and are barriers to care and treatment. Endemic gender based violence, driven by deep inequalities, creates conditions where women are up to three times more likely than men to become infected with HIV [1]. HIV disparities are even greater when examined by gender and sexual minority status. According to global meta-analyses, men who have sex with men (MSM) and transgender women (TW) experience 19 and 49 times the risk of HIV infection, respectively, compared to the general population of reproductive age adults [2, 3]. MSM and TW commonly face family rejection as well as social and cultural stigma and discrimination. These factors not only act as facilitators of the HIV epidemic but are often exacerbated by the criminalization of same-sex behaviors [4–6]. The effects of negative attitudes about gender equality and towards MSM, TW, and other gender and sexual minorities (GSM) held by policymakers (e.g. legislators and members of government ministries), health practitioners, and program staff are far reaching and contribute to the persistence of global HIV epidemics [7, 8]. For example, less than half of all countries included any reference to transgender people in their 2014 national HIV plans [9].

Gender- and sexuality-related stigma is often a hypothesized reason for the systematic global underinvestment in HIV programs that serve MSM and TW [8, 10–13]. Stigma-related marginalization of GSM is a likely explanation, given that data demonstrating the disproportionate burden faced by MSM around the world have been available for nearly two decades. The public health implications and human rights impact of laws and policies that perpetuate attitudes of stigma and discrimination need to be underscored as barriers to quality healthcare for MSM, TW and other GSM [5].

At service delivery levels, GSM’s negative experiences with health professionals erode trust and communication while seeking health services [14]. Providers are rarely expected to gain competency in communicating with and treating GSM patients, resulting in mislabeling and maltreatment [15, 16]. Other times, GSM are turned away entirely; or, due to the fear of stigmatizing treatment, GSM delay seeking care [17, 18]. For example, recent evidence drawn from an online sample of 2,981 MSM confirms significant associations between anticipation of homophobia and not seeking HIV prevention services [19].

Research findings suggest that attitudes around the world toward GSM are changing. In a survey of 37,653 citizens of 39 countries, Horowitz et al. found that acceptance of homosexuality increased by at least five percentage points in one-third of countries between 2007 and 2013 [20]. Still, more than 60 percent of respondents in El Salvador and Kenya and over 90 percent of respondents in Indonesia, Uganda, Ghana, and Nigeria indicated in 2013 that they believed homosexuality should not be accepted by society.

Increasing acceptance of GSM among policymakers, program staff, and health providers is a crucial step toward ensuring better access and uptake of HIV services. According to leading social psychologists, Zanna and Rempel, attitudes are the product of cognition (i.e., information and beliefs), affect (i.e., emotions), and past experiences [21, 22]. Efforts to create short-term changes in attitudes by way of sensitization workshops or training have been shown to result in positive changes with moderate consistency [23–26]. However, the effects of interventions on attitudes typically decay over time [27]. The distinction between short-term and long-term changes after such interventions is important for understanding how frequently training may be needed. Yet, long-term changes, also referred to as the attitude durability, are rarely measured experimentally.

The purpose of this manuscript is to report on the evaluation of the Gender & Sexual Diversity (GSD) Training that was developed by the Health Policy Project (HPP), an entity funded via the United States Agency for International Development (USAID) and the President’s Emergency Plan for AIDS Relief (PEPFAR, established in 2003). In coordination with a U.S. government interagency team comprised of members of the PEPFAR Key Populations Working Group and the PEPFAR Gender Technical Working Group, HPP offered the required training to PEPFAR-funded country teams. The curriculum was designed to help country programs understand and address the needs of GSM communities in the context of HIV programming and U.S. workplace policies on non-discrimination. Funding for the training was provided by PEPFAR via USAID.

### GSD training

With the recognition that addressing norms and attitudes related to gender and sexual orientation are important components of gender integration, the Gender & Sexual Diversity training focused on how gender and sexuality affect all people. The training aimed to (1) educate participants on terminology, U.S. government policies, and workplace expectations related to gender and sexual diversity; (2) sensitize participants to the needs of GSM, including as beneficiaries of HIV and other health programs; (3) provide examples and recommendations for meaningfully engaging GSM beneficiaries when developing and implementing programs; and (4) connect participants with local and regional resources on gender and sexual diversity issues. Using Zanna and Rempel’s conceptualization of attitudes, the training modules were designed to foster attitude change by impacting cognition and affect, and linking those to past experiences [28]. The purpose of the evaluation was to assess the training’s efficacy in changing attitudes toward GSM and determine its value as a tool to foster more effective key population programming.

The GSD Training curriculum was facilitated over the course of one full work day and contained four modules. Each module was designed to be highly engaging, employing diverse pedagogies that included slide presentations, interactive exercises, discussions and break out groups, and confidential electronic audience response systems—all with the intent of addressing components that shape attitudes (i.e., cognitions, affect, and past experiences).

Modules 1 and 2 provided information designed to impact cognition and opportunities to reflect on past experiences. Module 1 topics included: (1) epidemiological, financial, and human rights arguments for reaching and engaging GSM and increasing access to appropriate health services; (2) existing legal protections for U.S. government staff against employment discrimination based on sexual orientation and gender identity; (3) gender norms and their impact on health; and (4) the Updated PEPFAR Gender Strategy. Module 2 introduced concepts and terminology such as biological sex, gender expression, gender identity, and sexual orientation, and included activities that dealt with language, discrimination, and personal advocacy. Module 3 was focused on affect and included a panel of two members of local LGBT communities and one human rights advocate who each shared their story, describing what they believed to be the key challenges, success stories, and priorities for engaging with GSM in that country. Module 4 featured a discussion to build a shared understanding of the importance of meaningful engagement with GSM and an activity to illustrate meaningful engagement in action. This module was designed to build on potential positive attitude changes and promote positive behavioral intentions [29].

The GSD training was developed by context experts and training specialists at HPP in collaboration with members of the PEPFAR Key Populations Working Group and the PEPFAR Gender Technical Working Group. It was designed specifically for PEPFAR staff and their country-level implementing partners. This diverse audience spanned nationalities, languages, generations, religious and political beliefs, as well as educational backgrounds. Particular care was given to developing a curriculum that was flexible enough to be used across cultures.

While the curriculum remained the same in each country to ensure consistent messaging, facilitators met with local PEPFAR staff and local GSM community members prior to each training to discuss and prepare for the specific country context. GSM community members served as panelists during Module 3, during which they presented on personal and professional experiences of GSD in their country. When possible, GSM community members attended the entire training, joining other participants in conversations about GSD concepts and meaningful engagement. The final training manual and materials are available online at http://www.healthpolicyproject.com/index.cfm?ID=GSDTraining.

## Methods

**Fig 1:**
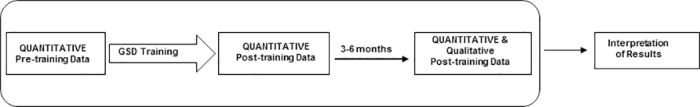
Study design for gender and sexual diversity training.

This independent outcome evaluation was approved by the ethics review board at Health Policy Project. All data were received anonymously for analysis by TP, CP, and BASN. The study was conducted using an embedded mixed methods pre-test/post-test design (Fig 1) in which qualitative data were collected to supplement the quantitative data [30]. Quantitative survey data were collected immediately before, immediately after, and three months following the GSD Training. Qualitative data were collected three to six months after the training to provide depth and context for the quantitative results.

### Quantitative

#### Participants

The GSD Training was offered to PEPFAR staff and program implementers in 38 countries. Trainings were open to other interested U.S. government staff and local stakeholders at the discretion of each PEPFAR country team. Each training lasted one day; and trainings were held multiple times within each country in order to accommodate the maximum number of participants. A total of 2,825 people in 38 countries received the PEPFAR GSD Training. Of these, 2,715 individuals in 33 countries participated in the training where a formal course evaluation was possible. Among these individuals, 1,766 people provided informed consent and valid data for a participation rate of 65%. Sample sizes for each included country are listed in Table 1. Data were excluded from five countries where trainings took place: Cameroon, Cote d’Ivoire, Honduras, Haiti, and Democratic Republic of Congo (DRC). In Cameroon, only pre-test data were collected using a pilot version of the questionnaire. In Cote d’Ivoire and Honduras, technical difficulties precluded data collection; and translation inconsistencies between pre- and post-test questionnaires invalidated data from Haiti and DRC.

**Table 1:** Number of gender and sexual diversity training participants by country.

Countries (n = 33)	Number of Pre/Post Participants	Number of Follow-up Participants
Angola	25	9
Barbados	32	6
Botswana	47	18
Burma	21	15
Cambodia	24	21
Dominican Republic	16	8
Ethiopia	63	17
Ghana	87	27
Guatemala	19	14
Guyana	79	31
India	32	17
Jamaica	16	0
Kazakhstan	21	13
Kenya	173	68
Kyrgyz Republic	17	8
Lesotho	30	6
Malawi	50	14
Mozambique	64	17
Namibia	61	15
Nicaragua	17	9
Nigeria	81	44
Papua New Guinea	34	0
Rwanda	75	25
South Africa	97	21
Swaziland	42	16
Tajikistan	20	7
Tanzania	55	32
Thailand	32	0
Uganda	174	17
Ukraine	19	9
Viet Nam	105	0
Zambia	81	42
Zimbabwe	57	30
**TOTAL**	**1766**	**576**

#### Procedures

Prior to data collection activities, participants were asked to provide informed consent for their de-identified data to be used for evaluation. Data from training participants who did not provide consent were destroyed and excluded from analyses. Training participants used individual handheld devices to indicate consent and to complete a brief set of questions measuring attitudes towards GSM and gender variance immediately prior to the training and immediately after the training. The devices transmitted data directly into Turning Point 5 software (Turning Technologies, Youngstown, OH) without being visible to participants. Use of individual handheld devices allowed for confidential linking of pre- and post-training data. Due to technology limitations, training participants in Barbados and Nicaragua used paper versions of the pre- and post-training questionnaires which were later manually entered into the software. Participants who consented to the use of their data recorded their participant number on each questionnaire so that data could be linked. Data were electronically stored on a password, encrypted cloud-based folder that was maintained by HPP. These data storage procedures ensured that the assessment information remained confidential and that only authorized personnel had access to it. Evaluation results are presented as aggregated data and no individual information is shared. While the PEPFAR training was required, consenting to have responses analyzed (i.e., participate in the evaluation) was a voluntary process.

Three months after the training, participants who provided consent were sent an email with a personalized link to the follow-up questionnaire. One reminder email was sent to non-responders approximately one month after the initial email with a second reminder being sent no less than two weeks apart for those who had not responded. Of the 1,766 initial participants, 576 (33%) responded to the 3-month follow-up survey. At no time did the evaluator have access to any identifying information for any of the participants. The evaluation was determined to be exempt by the ethical review committee at Palladium and a non-research determination (NRD) was approved by the Centers for Disease Control and Prevention (CDC).

#### Measures

Demographic data included gender (men, women, neither/both), age, and professional role (PEPFAR staff, non-PEPFAR U.S. government staff, program implementers, and other). Attitudes toward GSM were measured using an 8-item scale adapted from the 25-item scale, Support for Lesbian and Gay Human Rights [31]. The new scale had high internal consistency with a Cronbach alpha of 0.82. Attitudes toward gender variance were measured using a 6-item scale adapted from the 32-item Gender and Transgenderism Scale [32]. This new scale was also internally consistent with a Cronbach alpha of 0.83. Exploratory factor analysis indicated that a two-factor solution fit the data well; and item loadings were consistent with the two identified scales. Both measures used a 5-point Likert scale with response options ranging from strongly agree (1) to strongly disagree (5). Items for which agreement indicated a more negative attitude were reversed coded for aggregate analyses. Higher scale scores represented more negative attitudes toward GSM and gender variance.

In addition to these scales, participants were asked two questions to assess their self-efficacy for working with GSM: “I can list several ways I could take action to reduce stigma and discrimination against gender and sexual minorities in my workplace;” and “I can name at least two local organizations OR activists in my country that are supporting the health and human rights of gender and sexual minorities.” The number of measures that could be included was limited by the need to ensure that data collection was brief (i.e. only a few minutes) and did not delay or prolong the training activities.

#### Analysis

Data were analyzed using STATA version 13.1 (StataCorp, College Station, TX) and reported only for GSD trainees who provided informed consent. Frequencies were calculated for each of the demographic characteristics. For each of the scale items, the proportion of participants who agreed and strongly agreed was calculated. Next, for each participant, the overall score for each scale was taken as the average of the individual questions (after reverse coding of appropriate items). Where possible, the pre-training and post-training scores were linked by individual unique participant identifiers. Not every person who completed the pre-training survey remained for the post-training survey; thus, the sample size for the post-training scores is lower than for the pre-training scores. In addition, not every respondent to the emailed follow-up survey provided their participant identifier. Therefore, it was not possible to link data for all participants at all three time periods. The average scale scores were used to compare differences across time periods. The paired student t-test was used to determine statistical significance at p-values ≤ 0.05 for comparisons between pre- and post-training scores. The loss of more than two-thirds of the sample between pre-training (n = 1766) and follow-up (n = 576) precluded meaningful statistical comparison of the aggregate data at these two time points. However, a sub-analysis was conducted of data from the participants (n = 211) who could be linked at pre-training, post-training, and follow-up.

We fitted ordinal logistic regression models with random effects for participants and countries, and adjusted for participants’ age and gender [33]. These models were applied to the linked pre- and post-training data. Additionally, we applied the same models to data from the 211 participants who could be linked across all three time points. The distribution of the missing data across countries, age, and gender indicated that missing data were randomly distributed across these variables. We therefore conducted the regression analyses assuming responses were missing at random.

### Qualitative

#### Participants

Follow-up surveys completed three months after the training included an open-ended question asking respondents to describe any changes they had made in their work which may have been related to participation in the training. In addition, 29 qualitative key informant interviews were conducted in 21 countries approximately three to six months after the training. These interviews were designed to identify any perceived changes in the PEPFAR country team’s relationship with GSM communities after participation in the training. Key informants were purposively selected based on engagement in the training and familiarity with GSD issues. All key informants had attended the GSD training in their country, either as a community panelist or PEPFAR participant. For each country, the GSD training team identified a community panelist who had a strong understanding of the community and were present and engaged during the training. Each GSD training team also identified a PEPFAR staff member who attended the training and was engaged and familiar with the topic of GSD in the context of that country. These 29 individuals verbally consented to participate in the key informant interviews.

Twenty-one interviews were conducted with community members who had participated on the panel during the training. Eight interviews were conducted with PEPFAR staff who attended the training. Staff turnover and busy schedules limited availability of PEPFAR staff for follow-up interviews.

#### Procedures

Key informant interviews were conducted by Skype or telephone and lasted approximately 30 minutes. A semi-structured guide was used to organize the interview. Community members were asked to describe their role in the community and their relationship with PEPFAR, including receipt of PEPFAR funding, and to discuss whether they noticed any differences in local PEPFAR programming or community engagement after the training. PEPFAR staff were asked to describe their programming, in particular with key populations (i.e., MSM, transgender persons, people who inject drugs, and sex workers), and to discuss whether they noticed any differences in the workplace after the GSD Training. Interviews were led by trained staff and detailed notes were taken by a separate note-taker. Post interview field notes were typed by both the interviewer and note-taker.

#### Analysis

Thematic analysis was used for the qualitative data [34]. The interview notes and short, open-ended responses were analyzed separately by two coders who did not participate in any data collection activities. Each coder read all of the qualitative data for recurring themes and patterns, then sorted the text by theme. Memos were used to track the analytic process. Any differences in noted themes were discussed with the lead investigator until consensus was reached. Themes were then grouped by topical area based on research questions described above.

## Results

### Demographics

Participant demographics are listed in Table 2. Of the 1,766 participants in the study, 1,738 (98%) had responses for the pre-training survey and 1,429 (81%) contributed responses to the post-training survey. Of the 1,738 respondents with pre-training survey data, 46% (n = 790) were aged 39 years or younger, 48% (n = 841) were 40 years or older and 6% (n = 107) were missing age data. Fifty-three percent (n = 916) identified as women, 39% (n = 670) identified as men, 3% (n = 52) selected “neither/both” and 6% (n = 100) were missing gender data. Only 1,638 participants provided data on professional role. Most respondents were PEPFAR staff (53%, n = 892) or program implementers (32%, n = 539). The demographic distribution of the participants in the follow-up survey did not change.

**Table 2:** Demographics of training participants.

Demographics	Percent
**Age group (n = 1738)**	
≤39 years	46%, (n = 790)
≥40 years	48% (n = 841)
**Gender (n = 1738)**	
Women	53% (n = 916)
Men	39% (n = 670)
Neither/Both	3% (n = 52)
**Professional role (n = 1683)**	
PEPFAR Staff	53% (n = 892)
Program Implementers	32% (n = 539)
Other	9% (n = 151)
Non-PEPFAR USG Staff	6% (n = 101)

### Attitude changes by item

Overall, attitudes toward GSM and gender variance were more positive after the training compared to before the training. Item analysis within each scale revealed variability in the amount of change from baseline to immediately post-training (Table 3). The largest change in attitude toward GSM took place in responses to the following two questions: 1) “All people should be able to have any kind of consensual sex in private without being fined or arrested.” Agreement with this statement rose from 73% to 84%. 2) “Gender and sexual minorities should be allowed to express their opinions in public as long as they don’t offend most people.” Agreement with this statement rose from 65% to 75%. The vast majority of participants before (91%) and after (95%) the training agreed that no one should experience job discrimination because of their sexual orientation. Agreement that same sex couples should be allowed to marry was reported by 43% of participants prior to completing the training; and 48% agreed with this statement after the training. The least amount of change was seen in response to the statement: “It is okay for a newspaper to publicize that a person is a gender or sexual minority without that person’s permission.” Eight percent of participants agreed with the statement before the training; while 6% agreed after the training.

**Table 3:** Percent of respondents who agree and strongly agree with statements.

Attitudes toward Gender and Sexual Minorities^**ǂ**^	Percent	
	Pre	Post
1. No one should experience job discrimination because of their sexual orientation (n = 1,281)*	91%	95%
2. All people should be able to have any kind of consensual sex in private without being fined or arrested. (n = 1,210)*	73%	84%
3. Gender and sexual minorities should be allowed to express their opinions in public as long as they don’t offend most people. (n = 1,265)*	65%	75%
4. Gender and sexual minorities should be allowed to be school teachers. (n = 1,268)*	64%	74%
5. Same sex couples should be able to attend workplace social events together as partners (n = 1,246)*	63%	72%
6. Same sex couples should be legally permitted to marry. (n = 1,262)*	43%	48%
7. Policies that guarantee equal rights to gender and sexual minorities are bad for society. (n = 1,215)^R^*	16%	12%
8. It is okay for a newspaper to publicize that a person is a gender or sexual minority without that person’s permission. (n = 1,300)^R^*	8%	6%
Attitudes toward Gender Variance Scale*		
1. People are either men or women.(n = 1,283)^R^*	56%	37%
2. I am comfortable with masculine women. (n = 1,261)*	63%	71%
3. A man should be able to dress like a woman, if he chooses. (n = 1,204)*	49%	61%
4. I am comfortable with feminine men. (n = 1,276)*	57%	67%
5. I am comfortable working with feminine men. (n = 1,134)*	72%	80%
6. A woman should be able to present herself as a man in public, if she chooses. (n = 1,272)*	53%	65%
**Self-Efficacy**		
1. I can list several ways I could take action to reduce stigma and discrimination against gender and sexual minorities in my workplace. (Pre/Post: n = 1,257; Follow-up: n = 568)*	73%	95%
2. I can name at least two local organizations or activists in my country that are supporting the health and human rights of gender and sexual minorities. (Pre/Post: n = 1,197; Follow-up: n = 573)*	69%	88%

^R^Item was reverse coded when calculating the scale score.

^ǂ^Scales ranged from 1 = strongly agree to 5 = strongly disagree.

*Statistically significant, p< 0.05.

The largest change in attitude toward gender variance was seen in response to the question about whether people are either men or women (intended to imply a denial of non-binary gender identities), with a decrease from 56% to 37% who agreed with this statement. The majority of participants were comfortable with masculine women before (63%) and after (71%) the training. A smaller proportion of participants were comfortable with feminine men as compared to masculine women, both before (57% vs 63%, respectively) and after (67% vs 71%, respectively) the training. However, most participants were comfortable working with feminine men before (72%) and after (80%) the training. The smallest proportion of participants agreed that a man should be able to dress like a woman, both before (49%) and after (61%) the training.

### Attitude change by participant characteristic and scale scores

Table 4 depicts aggregate scale scores at baseline, immediately after the training, and at the 3-month follow-up period. Baseline scores were higher (indicating more negative attitudes) towards gender variance as compared to gender and sexual minorities (mean score 2.7 and 2.1, respectively). In the unadjusted, bivariate comparisons depicted in the table, overall scale scores demonstrated significantly less negative attitudes toward both GSM and gender variance post-training compared with pre-training. This change was consistent across gender and age. The same or lower scores were observed in both scales when comparing post-training and follow-up results.

**Table 4:** Average scale scores from pre-training, post-training, and 3–6 month follow-up.

Training Participant Characteristics	Average Scale Scores^ǂ^			Change in Scores	
	Pre	Post	Follow-up^˄^	Pre to Post	Pre to Follow-up
**Attitudes Toward Gender and Sexual Minorities Scale**					
**Gender**					
Men	**2.2** (n = 638)	**1.9** (n = 523)	**1.8** (n = 234)	−0.3*	−0.4
Women	**2.1** (n = 877)	**1.8** (n = 714)	**1.7** (n = 326)	−0.3*	−0.4
**Age group**					
≤39 years	**2.1** (n = 731)	**1.9** (n = 600)	**1.8** (n = 274)	−0.2*	−0.3
≥40 years	**2.1** (n = 784)	**1.8** (n = 637)	**1.8** (n = 286)	−0.3*	−0.3
**All**	**2.1** (n = 1,515)	**1.9** (n = 1,237)	**1.8** (n = 560)	−0.2*	−0.3
**Attitudes Toward Gender Variance Scale**					
**Gender**					
Men	**2.9** (n = 636)	**2.4** (n = 521)	**2.2** (n = 235)	−0.5*	−0.7
Women	**2.6** (n = 873)	**2.2** (n = 711)	**2.1** (n = 325)	−0.4*	−0.5
**Age group**					
≤39 years	**2.6** (n = 729)	**2.3** (n = 597)	**2.1** (n = 273)	−0.3*	−0.5
≥40 years	**2.7** (n = 780)	**2.3** (n = 635)	**2.1** (n = 287)	−0.4*	−0.6
**All Participants**	**2.7** (n = 1,509)	**2.3** (n = 1,232)	**2.2** (n = 560)	−0.4*	−0.5

^ǂ^A lower score represents a more positive attitude toward sexual minorities.

*Statistically significant, p< 0.05.

^˄^Pre- and post-training data are linked, but follow-up data were not linked; therefore, no statistical tests were conducted with follow-up data.

On multivariable analyses adjusted for age and gender, the odds of higher scores (i.e. more negative) on the Attitudes Toward GSM scale were reduced by 73% from pre- to post-training [OR 0.27; 95%CI: 0.23, 0.33, p<0.0001] (Table 5: Model A). In this model, women had less negative attitudes than men [OR 0.56; 95%CI: 0.38, 0.82, p = 0.003], but there were no significant differences in scores by age. In an adjusted model that included only participants with linked data at all three time points (Table 5: Model B), the odds of higher scores on the Attitudes Toward GSM Scale were reduced by 76% from pre- to post-training [OR 0.24; 95%CI: 0.15, 0.39, p<0.0001] and 71% from pre-training to 3-month follow-up [OR 0.29, 95%CI: 0.18; 0.46, p<0.0001]. In this adjusted model, participants aged 40 years and older were more likely to have higher scores compared with those 39 and younger [OR 3.07; 95%CI: 1.2, 8.41, p = 0.029]; however, there were no significant differences in scores by gender.

**Table 5:** Multivariable ordinal logistic regression models of factors associated with attitudes toward gender and sexual minorities.

Covariate	aOR	95% CI	p-value
**Model A: Aggregated Pre/Post Data (n = 1766)**			
Post-training*	0.27	0.23, 0.33	<0.0001
Age > 40 years	1.04	0.71, 1.53	0.830
Woman*	0.56	0.38, 0.82	0.003
Neither/Both	0.51	0.16, 1.61	0.254
**Model B: Linked Pre-, Post-, Follow-up Data (n = 211)**			
Post-training*	0.24	0.15, 0.39	<0.0001
Follow-up*	0.28	0.18, 0.46	<0.0001
Age > 40 years*	3.07	1.12, 8.41	0.029
Woman	1.02	0.37, 2.84	0.962
Neither/Both	0.22	0.01, 6.70	0.383

*Statistically significant, p< 0.05

Similarly, Attitudes Toward Gender Variance scale scores were lower after the training compared with pre-training scores. On multivariable analyses adjusted for age and gender, the odds of higher scores (i.e. more negative attitudes toward gender variance) were reduced by 71% from pre- to post-training [OR 0.29; 95%CI: 0.24, 0.34, p<0.0001] (Table 6: Model A). In this model, women had lower scores (less negative attitudes) than men [OR 0.38; 95%CI: 0.26, 0.55, p<0.0001]; and older participants had higher scores (more negative attitudes) than younger ones [OR 1.49; 95%CI 1.04, 2.15, p = 0.031]. In an adjusted model with all three time points (Table 6: Model B), the odds of higher scores were reduced by 68% from pre- to post-training [OR 0.32; 95%CI: 0.21, 0.48, p<0.0001] and 79% from pre-training to 3-month follow-up [OR 0.21, 95%CI: 0.14; 0.33, p<0.0001]. There were no significant differences by age or gender.

**Table 6:** Multivariable ordinal logistic regression models of factors associated with attitudes toward gender variance.

Covariate	aOR	95% CI	p-value
**Model A: Aggregated Pre/Post Data (n = 1766)**			
Post-training*	0.29	0.24, 0.34	<0.0001
Age > 40 years*	1.49	1.04, 2.15	0.031
Woman*	0.38	0.26, 0.55	<0.0001
Neither/Both	0.20	0.07, 0.58	0.003
**Model B: Linked Pre-, Post-, Follow-up Data (n = 211)**			
Post-training*	0.32	0.21, 0.48	<0.0001
Follow-up*	0.21	0.14, 0.33	<0.0001
Age > 40 years	2.04	0.89, 4.64	0.090
Woman	0.76	0.33, 1.76	0.528
Neither/Both	0.06	0.03, 1.01	0.051

*Statistically significant, p< 0.05

Sensitivity analyses were conducted by fitting regression models that excluded participants with missing data at either pre- or post-training, and the results did not change significantly. In addition, analyses were conducted excluding participants who answered less than half of the questions on each scale. Again, the results did not change significantly.

### Changes in self-efficacy and self-reported behavior

The largest change from baseline took place in self-efficacy measures (Table 3). Self-efficacy for reducing GSM stigma and discrimination in the workplace rose from 73% to 95% post-training and remained high at 95% three months after the training (n = 568, data not shown). The proportion of participants who could identify local organizations or activists who support GSM human rights increased from 69% to 88% and was maintained at 87% three months after the training (n = 573, data not shown). These findings were compared with the subset of participants for whom linked pre-, post-, and 3-month follow-up data were available for these items (n = 192). No differences were found with the following exception: 96% of this subset of participants were able to identify local organizations or activists immediately after the training and 89% were able to do so three months following the training.

Approximately half of the respondents (53%) on the follow-up survey reported having done something differently in their workplaces or programs following the GSD training; and 311 provided specific comments about what they had done differently. Participants described actions that fell into three broad categories in order of frequency: (1) treating GSM with greater acceptance or respect; (2) educating others; and (3) adapting or modifying GSM programming. Table 7 includes illustrative quotes from participant responses.

**Table 7:** Illustrative quotes of participant changes after gsd training.

Categories of Change	Illustrative Quotes
**Treated GSM with greater acceptance**	*More confidently expressing views and having empathy towards the cause of TG* [transgender] *individuals*.
	*Try to be more cautious and respectful when making comments in the office and using some terms that might make others uncomfortable*.
	*My behavior has changed with regard to gender and sex*. *We are now all the same whether gay*, *lesbian*, *all the same*. *And who am I to judge other people*.
**Educated others about GSD**	*Sensitized Health Care workers on the need for health services delivery to be sensitive to the needs of people with different sexual orientations*.
	*Shared the handouts and materials with staff in my workplace*
	*Sharing the knowledge [of] what I learn[ed] from this training with my colleagues*, *as a result; I just found out one of my colleagues*, *who really hated the transgender women*, *changed his mind and understands the nature (actually mind*, *biology and life) of transgender women*. *I do not hear bad comments anymore from him*.
**Improved programming for GSM**	*I am doing advocacy with MOH for best services for key population*.
	*Engaging and working closely with LGBT organisations and activists to support the work related to LGBT people*.
	*The HIV care & treatment program I manage is now providing services to the LGBTI community*.

### Qualitative findings on community engagement and programming

Qualitative interviews allowed participants to describe their experience of the training and any changes they noticed in the PEPFAR teams’ work with GSM as a consequence of the training. Every person interviewed (n = 29) described the community panel as the most compelling part of the training. Community members felt empowered by their experiences on the panel, and PEPFAR staff felt that people connected with the experiences of GSM in a deeper way after interacting with panelists. A community member stated, “S*o that it’s not just top down*, *it was bottom-up*. *It was an opportunity for meaningful engagement to take place*.*”* A PEPFAR staff member agreed, “*I think the panel was really a highlight in having an opportunity to see that these are real people and they aren’t being pushed to the side*.”

Most PEPFAR staff and all community members interviewed felt that the training was valuable and recommended that it take place regularly and reach a wider audience. However, few could link the training to a specific change in PEPFAR community engagement or programming for GSM. Several PEPFAR respondents pointed out that the training took place after their annual planning cycle; therefore, it would be another year before they would know if there was an impact. Some noted that 3–6 months was not enough time to determine if there had been a change. Other PEPFAR staff felt that their team was already appropriately engaging GSM in their work; therefore, changes weren’t needed. A few PEPFAR staff members noted that meeting the panelists provided an opportunity to engage with a community organization that the team had not known before. The majority of community members interviewed had little familiarity with the PEPFAR program planning process. However, a few community members noted new or more engaged relationships with PEPFAR staff or implementers in the 6 months since the training. For example, a community member stated, “*Previously there were organizations that partners would not engage with but are now engaging with*. *The training itself opened doors to different partners that we work with*. *These people are resources*. *The training itself is something that made people think out of the box*.”

## Discussion

The Joint United Nations Programme on HIV/AIDS (UNAIDS) has set ambitious targets to end the AIDS epidemic by 2030. These targets include ensuring that 90% of people living with HIV know their HIV status; 90% of people living with HIV receive antiretroviral therapy (ART); and 90% of people receiving ART have viral suppression [35]. In support of these “90-90-90” targets, PEPFAR 3.0 committed to a human rights agenda that focuses on ending stigma and discrimination against key populations, including GSM, in order to reach them with HIV testing, prevention, and treatment services [36].

Effective engagement of GSM in HIV services requires a greater understanding and acceptance of GSM among policymakers, program implementers, and service providers. Both qualitative and quantitative findings at post and follow-up assessments of this one-day PEPFAR GSD training indicate improvements in attitudes toward GSM as compared to baseline findings. Qualitative data suggest that many of the training participants translated these positive attitude changes into action in the workplace.

By challenging negative beliefs about GSM with factual information, emotionally engaging participants during the community panel, and encouraging positive behavioral intentions through role plays and practical exercises, the PEPFAR GSD training addressed key components of attitude formation [21]. A pre-training to post-training decrease in negative attitudes was not surprising for a one-day training that received high level support from in-country leadership and in which facilitators set clear norms for GSD respect and tolerance. However, the effects of attitude change interventions typically decay with time [27]. Unlike most interventions, more positive attitudes toward GSM not only persisted 3–6 months after that training but improved even further. It is possible that attendance by most, if not all, PEPFAR staff at a site may have helped to reset the norms of the workplace, setting up a self-reinforcing environment of tolerance and expectation of respect.

Several study limitations exist. The evaluation had a low response rate (33%) to the three-month follow-up questionnaire. However, this response rate falls within the range of 24–48% that is typical of many email surveys [37, 38]. A limited subsample (12%) provided data that could be linked at all three time points (pre-, post-, and 3–6 months following the training); and we observed improvements in attitudes within this sub-sample that exceeded those of the overall three-month respondents. There were also a limited number of PEPFAR staff interviews even though they were the intended audience of training. The possibility that those who did not respond had the most negative attitudes must also be considered; and generalizability of the finding are limited. While the subsample with linked data at all three time points may not represent the full sample, the findings suggest the possibility of an enduring positive change in attitudes towards GSM. The study was limited to self-reported data. We were unable to verify the behavioral changes reported by participants in the qualitative data, and our measures for self-efficacy had not been validated in prior studies. The need to limit the questionnaires to no more than five minutes precluded the inclusion of a social desirability scale. Therefore, we cannot assess how much the results may have been impacted by social desirability bias. Future studies would benefit from assessing this important source of bias.

Despite limitations, triangulation of all data sources (attitude scales, open-ended survey responses, and key informant interviews) suggests that the one-day PEPFAR GSD training was successful in changing attitudes toward GSM. Importantly, these changed attitudes were sustained for months after the training, and attendees noted positive changes in the workplace and, to a lesser extent, HIV programming. It is likely that repeated exposure via annual or semi-annual refresher trainings would be needed to sustain positive attitudes long-term as PEPFAR staff come and go based on the rotating nature of U.S. government international positions. As the first study to report GSD attitude change among a global sample of almost 1,800 individuals from 33 countries, the findings provide strong evidence for the capacity of carefully crafted GSD trainings to effect change. More longitudinal research is needed to better understand how long these changes in attitude may persist and to determine the appropriate intervals for refresher trainings. Given the great time demands on PEPFAR staff and implementers, future research should also explore whether alternative training modalities (e.g., shorter trainings, online trainings) would have similar outcomes.
